# Supplementary material for: Content, Structure and Delivery Characteristics of Yoga Interventions for Managing Rheumatoid Arthritis: A Systematic Review Protocol
Source: Int J Environ Res Public Health. 2022 May 17;19(10):6102. doi: 10.3390/ijerph19106102 (PMC9140818; doi:10.3390/ijerph19106102)
Supplement: Supplementary file 1 [file ijerph-19-06102-s001.zip › ijerph-1707164-supplementary.pdf]

Supplementary File S1: Search strategies

A Bibliography of Indian Medicine (ABIM)<Date of search: April 14, 2022>: 3 records

Search terms used: yoga and rheumatoid arthritis

Allied and Complementary Medicine (AMED) (Ovid) <1985 to April 14, 2022>: 8 records

1. exp Mind body medicine/
2. exp Yoga/
3. exp Meditation/
4. Mind body medicine*.mp.
5. (Yoga* or yogi*).mp.
6. Meditat*.mp.
7. (Asana* or Pranayam* or Dhyan* or Ashtanga or Bikram or Hatha or Iyengar or Kripalu or Kundalini or Vinyasa or Raja or Radja or Bhakti or Jnana or Kriya* or Karma or Yama or Niyama or Pratyahara or Dharana or Samadhi or Bandha or Mudra* or Chanda or Sivananda).mp.
8. 1 or 2 or 3 or 4 or 5 or 6 or 7
9. exp Arthritis rheumatoid/
10. ((rheumatoid or reumatoid or revmatoid or rheumatic or reumatic or revmatic or rheumat* or reumat* or revmarthrit*) adj3 (arthrit* or artrit* or diseas* or condition* or nodule*)).mp.
11. (Sjogren* adj2 syndrome).mp.
12. (sicca adj2 syndrome).mp.
13. still* disease.mp.
14. bechterew* disease.mp.
15. (caplan* adj2 syndrome).mp.
16. (felty* adj2 syndrome).mp.
17. (rheumatoid adj2 factor*).mp.
18. ((inflammatory or idiopathic) adj2 (arthritis or polyarthritis)).mp.
19. 9 or 10 or 11 or 12 or 13 or 14 or 15 or 16 or 17 or 18
20. (random* or factorial* or placebo* or assign* or allocat* or crossover*).tw.
21. (cross adj over*).tw.
22. (trial* and (control* or comparative)).tw.
23. ((blind* or mask*) and (single or double or triple or treble)).tw.
24. (treatment adj arm*).tw.
25. (control* adj group*).tw.
26. (phase adj (III or three)).tw.
27. (versus or vs).tw.
28. rct.tw.
29. RANDOM ALLOCATION/
30. DOUBLE BLIND METHOD/
31. placebos/
32. randomized controlled trials/
33. 20 or 21 or 22 or 23 or 24 or 25 or 26 or 27 or 28 or 29 or 30 or 31 or 32
34. 8 and 19 and 33

AYUSH Research Portal (Ministry of AYUSH, Government of India) <Date of search: April 14, 2022>: 6294 records

Medical system: yoga and naturopathy
Category: preclinical research, clinical research (Evidence grade - A, B, C) and fundamental research
Body system: musculoskeletal
Disease (English): Rheumatoid/seropositive arthritis (ICPC-L88, ICD-M05, M06, M08, M45)

Evidence grade-A:991 Evidence grade-B:1784 Evidence grade-C:3519

Complementary and alternative medicine (CAM-QUEST) < Date of search: April 14, 2022>: 42 records

Searched for the following:
Disease pattern: pain (1155 results) to disease: chronic pain (209 results) to therapy: mind body medicine (75 results) to study design: randomized trial (29 results)

Searched for the following:

Therapy: Mind-body medicine (3708 results) to disease pattern: Musculoskeletal-/Connective tissue system (388 results) to disease: Arthritis (24 results) to study design: Randomized trial (13 results)

CINAHL (EBSCOHost) <1994 to April 14, 2022 >: 455 records

S1 (MH "Mind Body Techniques+")
S2 TX Yoga* or yogi*
S3 TX "Mind body therap*"
S4 TX Meditat*
S5 TX Asana* or Pranayam* or Dhyan* or Ashtanga or Bikram or Hatha or Iyengar or Kripalu or Kundalini or Vinyasa or Raja or Radja or Bhakti or Jnana or Kriya* or Karma or Yama or Niyama or Pratyahara or Dharana or Samadhi or Bandha or Mudra* or Chanda or Sivananda
S6 TX S1 or S2 or S3 or S4 or S5
S7 (MH "Arthritis, Rheumatoid+")
S8 TX Rheumatoid arthritis
S9 TX Sjogren* syndrome
S10 TX Sicca syndrome
S11 TX Still* disease
S12 TX Bechterew disease
S13 TX Caplan syndrome
S14 TX Felty* syndrome
S15 TX rheumatoid N2 factor*
S16 TX (inflammatory OR idiopathic) N2 (arthritis or polyarthritis)
S17 S7 or S8 or S9 or S10 or S11 or S12 or S13 or S14 or S15 or S16
S18 TX randomized controlled trials
S19 MH double-blind studies
S20 MH single-blind studies
S21 MH random assignment
S22 MH pretest-posttest design
S23 MH cluster sample
S24 TI (randomised or randomized)
S25 AB (random*)
S26 TI (trial)
S27 (MH (sample size) and AB (assigned or allocated or control))
S28 MH (placebos)
S29 PT (randomized controlled trial)
S30 AB (control W5 group)
S31 MH (crossover design)
S32 MH (comparative studies)
S33 AB (cluster W3 RCT))
S34 S18 or S19 or S20 or S21 or S22 or S23 or S24 or S25 or S26 or S27 or S28 or S29 or S30 or S31 or S32 or S33
S35 ((MH animals+ or MH animal studies or TI animal model*) not MH human)
S36 S34 not S35
S37 S6 and S17 and S36

Cochrane Central Register of Controlled Trials (CENTRAL) <1996 to April 14, 2022>: 585 records

#1 MeSH descriptor: [Mind-Body Therapies] explode all trees
#2 Meditation (Word variations have been searched)
#3 ((yoga*)) (Word variations have been searched)
#4 (Mind body therapies) (Word variations have been searched)
#5 (asana* or pranayam* or dhyan* or meditat* or ashtanga or bikram or hatha or iyengar or kripalu or kundalini or vinyasa or raja or radja or bhakti or jnana or kriya* or karma or yama or niyama or pratyahara or dharana or samadhi or bandha or mudra* or chanda or sivananda)
#6 #1 or #2 or #3 or #4 or #5
#7 MeSH descriptor: [Arthritis, Rheumatoid] explode all trees
#8 (Sjogren* adj2 syndrome).mp. (Word variations have been searched)
#9 (Sicca adj2 syndrome).mp. (Word variations have been searched)
#10 Still* disease.mp. (Word variations have been searched)
#11 Bechterew* disease.mp. (Word variations have been searched)
#12 (Caplan* adj2 syndrome).mp. (Word variations have been searched)
#13 (Felty* adj2 syndrome).mp. (Word variations have been searched)
#14 (rheumatoid adj2 factor*).mp. (Word variations have been searched)
#15 ((inflammatory or idiopathic) adj2 (arthritis or polyarthritis)).mp. (Word variations have been searched)
#16 #7 or #8 or #9 or #10 or #11 or #12 or #13 or #14 or #15 (Word variations have been searched)
#17 MeSH descriptor: [Randomized Controlled Trial] explode all trees
#18 MeSH descriptor: [Controlled Clinical Trial] explode all trees
#19 placebo* (Word variations have been searched)
#20 ("randomised clinical trial") (Word variations have been searched)
#21 (trial*) (Word variations have been searched)
#22 MeSH descriptor: [Randomized Controlled Trials as Topic] explode all trees
#23 #17 or #18 or #19 or #20 or #21 or #22 (Word variations have been searched)
#24 #6 and #16 and #23 (Word variations have been searched)

EMBASE (Ovid) <1974 to April 14, 2022 >: 303 records

1. exp alternative medicine/
2. exp yoga/
3. exp meditation/
4. Mind body therap*.mp.
5. (Yoga* or yogi*).mp.
6. Meditat*.mp.
7. (Asana* or Pranayam* or Dhyan* or Ashtanga or Bikram or Hatha or Iyengar or Kripalu or Kundalini or Vinyasa or Raja or Radja or Bhakti or Jnana or Kriya* or Karma or Yama or Niyama or Pratyahara or Dharana or Samadhi or Bandha or Mudra* or Chanda or Sivananda).mp.
8. 1 or 2 or 3 or 4 or 5 or 6 or 7
9. exp rheumatoid arthritis/
10. ((rheumatoid or reumatoid or revmatoid or rheumatic or reumatic or revmatic or rheumat$ or reumat$ or revmarthrit$) adj3 (arthrit$ or artrit$ or diseas$ or condition$ or nodule$)).mp.
11. (sjogren$ adj2 syndrome).mp.
12. (sicca adj2 syndrome).mp.
13. still$ disease.mp.
14. bechterew$ disease.mp.
15. (caplan* adj2 syndrome).mp.
16. (felty* adj2 syndrome).mp.
17. (rheumatoid adj2 factor*).mp.
18. ((inflammatory or idiopathic) adj2 (arthritis or polyarthritis)).mp.
19. 9 or 10 or 11 or 12 or 13 or 14 or 15 or 16 or 17 or 18

20. Randomized controlled trial/
21. Controlled clinical trial/
22. random*.ti,ab.
23. randomization/
24. intermethod comparison/
25. placebo.ti,ab.
26. (compare or compared or comparison).ti.
27. ((evaluated or evaluate or evaluating or assessed or assess) and (compare or compared or comparing or comparison)).ab.
28. (open adj label).ti,ab.
29. ((double or single or doubly or singly) adj (blind or blinded or blindly)).ti,ab.
30. double blind procedure/
31. parallel group*1.ti,ab.
32. (crossover or cross over).ti,ab.
33. ((assign* or match or matched or allocation) adj5 (alternate or group*1 or intervention*1 or patient*1 or subject*1 or participant*1)).ti,ab.
34. (assigned or allocated).ti,ab.
35. (controlled adj7 (study or design or trial)).ti,ab.
36. (volunteer or volunteers).ti,ab.
37. human experiment/
38. trial.ti.
39. 20 or 21 or 22 or 23 or 24 or 25 or 26 or 27 or 28 or 29 or 30 or 31 or 32 or 33 or 34 or 35 or 36 or 37 or 38
40. (random* adj sampl* adj7 (cross section* or questionnaire*1 or survey* or database*1)).ti,ab. not (comparative study/ or controlled study/ or randomi?ed controlled.ti,ab. or randomly assigned.ti,ab.)
41. Cross-sectional study/ not (randomized controlled trial/ or controlled clinical study/ or controlled study/ or randomi?ed controlled.ti,ab. or control group*1.ti,ab.)
42. (((case adj control*) and random*) not randomi?ed controlled).ti,ab.
43. (Systematic review not (trial or study)).ti.
44. (nonrandom* not random*).ti,ab.
45. Random field*.ti,ab.
46. (random cluster adj3 sampl*).ti,ab.
47. (review.ab. and review.pt.) not trial.ti.
48. we searched.ab. and (review.ti. or review.pt.)
49. update review.ab.
50. (databases adj4 searched).ab.
51. (rat or rats or mouse or mice or swine or porcine or murine or sheep or lambs or pigs or piglets or rabbit or rabbits or cat or cats or dog or dogs or cattle or bovine or monkey or monkeys or trout or marmoset$1).ti. and animal experiment/
52. Animal experiment/ not (human experiment/ or human/)
53. 40 or 41 or 42 or 43 or 44 or 45 or 46 or 47 or 48 or 49 or 50 or 51 or 52
54. 39 not 53
55. 8 and 19 and 54

MEDLINE (Ovid) <1946 to April 14, 2022 >: 104 records

1. exp Mind-Body Therapies/
2. mind body therap*.mp.
3. meditat*.mp.
4. (yoga* or yogi*).mp.
5. (Asana* or Pranayam* or Dhyan* or Ashtanga or Bikram or Hatha or Iyengar or Kripalu or Kundalini or Vinyasa or Raja or Radja or Bhakti or Jnana or Kriya* or Karma or Yama or Niyama or Pratyahara or Dharana or Samadhi or Bandha or Mudra* or Chanda or Sivananda).mp.
6. 1 or 2 or 3 or 4 or 5

7. exp Arthritis, Rheumatoid/
8. ((rheumatoid or reumatoid or revmatoid or rheumatic or reumatic or revmatic or rheumat* or reumat* or revmarthrit*) adj3 (arthrit* or artrit* or diseas* or condition* or nodule*)).mp.
9. (Sjogren* adj2 syndrome).mp.
10. (sicca adj2 syndrome).mp.
11. still* disease.mp.
12. bechterew* disease.mp.
13. (caplan* adj2 syndrome).mp.
14. (felty* adj2 syndrome).mp.
15. (rheumatoid adj2 factor*).mp.
16. ((inflammatory or idiopathic) adj2 (arthritis or polyarthritis)).mp.
17. 8 or 9 or 10 or 11 or 12 or 13 or 14 or 15 or 16
18. randomized controlled trial.pt.
19. controlled clinical trial.pt.
20. randomized.ab.
21. placebo.ab.
22. drug therapy.fs.
23. randomly.ab.
24. trial.ab.
25. groups.ab.
26. 18 or 19 or 20 or 21 or 22 or 23 or 24 or 25
27. exp animals/ not humans.sh.
28. 26 not 27
29. 6 and 17 and 28

Physiotherapy Evidence Database (PeDro) <from 1999 to April 14, 2022>: 13 records

Searched terms used: yoga and rheumatoid arthritis
13 records out of which 6 RCTs

PsycInfo (OVID) <1806 to April 14, 2022>: 3 records

1. exp Mind Body Therapy/
2. Mind-body therap*.mp.
3. exp Yoga/
4. (Yoga* or yogi*).mp.
5. exp Meditation/
6. Meditat*.mp.
7. (Asana* or Pranayam* or Dhyan* or Ashtanga or Bikram or Hatha or Iyengar or Kripalu or Kundalini or Vinyasa or Raja or Radja or Bhakti or Jnana or Kriya* or Karma or Yama or Niyama or Pratyahara or Dharana or Samadhi or Bandha or Mudra* or Chanda or Sivananda).mp.
8. 1 or 2 or 3 or 4 or 5 or 6 or 7
9. exp Rheumatoid Arthritis/
10. ((rheumatoid or reumatoid or revmatoid or rheumatic or reumatic or revmatic or rheumat* or reumat* or revmarthrit*) adj3 (arthrit* or artrit* or diseas* or condition* or nodule*)).mp.
11. (Sjogren* adj2 syndrome).mp.
12. (sicca adj2 syndrome).mp.
13. still* disease.mp.
14. bechterew* disease.mp.
15. (caplan* adj2 syndrome).mp.
16. (felty* adj2 syndrome).mp.
17. (rheumatoid adj2 factor*).mp.
18. ((inflammatory or idiopathic) adj2 (arthritis or polyarthritis)).mp.
19. 9 or 10 or 11 or 12 or 13 or 14 or 15 or 16 or 17 or 18
20. (Randomized Controlled Trial or Controlled Clinical Trial or Pragmatic Clinical Trial or Equivalence Trial or Clinical Trial, Phase III).pt.

21. Randomized Controlled Trial/
22. exp Randomized Controlled Trials/
23. "Randomized Controlled Trial (topic)"/
24. Controlled Clinical Trial/
25. Controlled Clinical Trials/
26. exp Clinical Trials/
27. "Controlled Clinical Trial (topic)"/
28. Randomization/
29. Random Allocation/
30. Double-Blind Method/
31. Double Blind Procedure/
32. Double-Blind Studies/
33. Single-Blind Method/
34. Single Blind Procedure/
35. Single-Blind Studies/
36. Placebos/
37. Placebo/
38. Control Groups/
39. Control Group/
40. (random* or sham or placebo*).ti,ab,hw.
41. ((singl* or doubl*) adj (blind* or dumm* or mask*)).ti,ab,hw.
42. ((tripl* or trebl*) adj (blind* or dumm* or mask*)).ti,ab,hw.
43. (control* adj3 (study or studies or trial* or group*)).ti,ab.
44. (Nonrandom* or non random* or non-random* or quasi-random* or quasirandom*).ti,ab,hw.
45. allocated.ti,ab,hw.
46. ((open label or open-label) adj5 (study or studies or trial*)).ti,ab,hw.
47. ((equivalence or superiority or non-inferiority or noninferiority) adj3 (study or studies or trial*)).ti,ab,hw.
48. (pragmatic study or pragmatic studies).ti,ab,hw.
49. ((pragmatic or practical) adj3 trial*).ti,ab,hw.
50. ((quasiexperimental or quasi-experimental) adj3 (study or studies or trial*)).ti,ab,hw.
51. (phase adj3 (III or "3") adj3 (study or studies or trial*)).ti,hw.
52. 20 or 21 or 22 or 23 or 24 or 25 or 26 or 27 or 28 or 29 or 30 or 31 or 32 or 33 or 34 or 35 or 36 or 37 or 38 or 39 or 40 or 41 or 42 or 43 or 44 or 45 or 46 or 47 or 48 or 49 or 50 or 51
53. 8 and 19 and 52

SPORTDiscus (EBSCOHost) <2004 to April 14, 2022>: 4 records

S1 SU Mind body therapy
S2 SU Yoga
S3 SU Meditation
S4 TX Mind body therap*
S5 TX Yoga* or yogi*
S6 TX Meditat*
S7 TX Asana* or Pranayam* or Dhyan* or Ashtanga or Bikram or Hatha or Iyengar or Kripalu or Kundalini or Vinyasa or Raja or Radja or Bhakti or Jnana or Kriya* or Karma or Yama or Niyama or Pratyahara or Dharana or Samadhi or Bandha or Mudra* or Chanda or Sivananda
S8 TX S1 or S2 or S3 or S4 or S5 or S6 or S7
S9 SU rheumatoid arthritis
S10 TX Rheumatoid arthritis
S11 TX Sjogren* syndrome
S12 TX Sicca syndrome
S13 TX Still* disease
S14 TX Bechterew disease
S15 TX Caplan syndrome

S16 TX Felty* syndrome
S17 TX rheumatoid N2 factor*
S18 TX (inflammatory or idiopathic) N2 (arthritis or polyarthritis)
S19 TX S9 or S10 or S11 or S12 or S13 or S14 or S15 or S16 or S17 or S18
S20 TX Randomized controlled trials
S21 SU Randomized controlled trials
S22 TX Double-blind studies
S23 TX Single-blind studies
S24 TX Random assignment
S25 TX Pretest-posttest design
S26 TX Cluster sample
S27 TX Placebos
S28 TX randomised or randomized
S29 TX random*
S30 TX trial*
S31 TX S21 or S22 or S23 or S24 or S25 or S26 or S27 or S28 or S29 or S30 or S30
S32 TX S8 and S19 and S31

Turning Research Into Practice (TRIP); < 2014 to April 14, 2022>: 251 records

(yoga* or "mind body therapies" or yogi* or asana* or pranayam* or dhyan* or meditation or meditate or ashtanga or bikram or hatha or iyengar or kripalu or kundalini or vinyasa or raja or radja or bhakti or jnana or kriya* or karma or yama or niyama or pratyahara or dharana or samadhi or bandha or mudra* or chanda or sivananda) and ("rheumatoid arthritis" or "sjogren* syndrome" or "sicca syndrome" or "still* disease" or "bechterew* disease" or "caplan* syndrome" or "felty* syndrome" or "rheumatoid factor" or inflammatory arthritis or idiopathic arthritis or inflammatory polyarthritis) and (randomised controlled trial or randomized controlled trial or controlled clinical trial* or clinical trial* or placebo* or random*)

Web of Science <1998 to April 14, 2022>: 78 records

#1 ALL= (yoga* or "mind body therap*" or meditation or yogi* or asana* or pranayam* or dhyan* or meditat* or ashtanga or bikram or hatha or iyengar or kripalu or kundalini or vinyasa or raja or radja or bhakti or jnana or kriya* or karma or yama or niyama or pratyahara or dharana or samadhi or bandha or mudra* or chanda or sivananda)
#2 ALL=("Rheumatoid arthritis" or "sjogren* syndrome" or "sicca syndrome" or "still* disease" or "bechterew* disease" or "caplan* syndrome" or "felty* syndrome" or rheumatoid factor or "inflammatory arthritis" or "idiopathic arthritis" or "inflammatory polyarthritis")
#3 ALL=(("randomized controlled trial" or "controlled clinical trial" or "clinical trial" or "clinical trials" or placebo* or "random allocation" or "double-blind method" or "single-blind method" or "cross-over studies"))
#4 (ALL=(("randomized controlled trial" or "controlled clinical trial" or "clinical trial" or "clinical trials" or placebo* or "random allocation" or "double-blind method" or "single-blind method" or "cross-over studies"))) and ALL=((randomised or randomized or randomisation or randomisation or placebo* or (random* and (allocat* or assign*)) or (blind* and (single or double or treble or triple))))
#5 #3 or #4
#6 #1 and #2 and #5

DART-Europe-e-theses portal <from 2005 to April 14, 2022>: 1 record

Search terms used: yoga and rheumatoid arthritis

EthOS <from 2009 to April 14, 2022>: 0 records

Search terms used: yoga and rheumatoid arthritis

OpenGrey (Data Archiving and Network Services) <from 1997 to April 14, 2022>: 0 records

Search terms used: Yoga and rheumatoid arthritis

ProQuest Dissertations and Theses <from 1902 to April 14, 2022>: 238 records

(yoga* or mind body therap* or yogi* or asana* or pranayam* or dhyan* or meditat* or ashtanga or bikram or hatha or iyengar or kripalu or kundalini or vinyasa or raja or radja or bhakti or jnana or kriya* or karma or yama or niyama or pratyahara or dharana or samadhi or bandha or mudra* or chanda or sivananda) and ("rheumatoid arthritis" or "sjogren* syndrome" or "sicca syndrome" or "still* disease" or "bechterew* disease" or "caplan* syndrome" or "felty* syndrome" or "rheumatoid factor" or inflammatory arthritis or idiopathic arthritis or inflammatory polyarthritis) and (randomised controlled trial or randomized controlled trial or controlled clinical trial* or clinical trial* or placebo* or random* or trial*)
Filters applied: Rheumatoid arthritis or arthritis
